# Serum glycolipids mediate the relationship of urinary bisphenols with NAFLD: analysis of a population-based, cross-sectional study

**DOI:** 10.1186/s12940-022-00945-w

**Published:** 2023-01-02

**Authors:** Jia Peng, Lei-Lei Du, Qi-Lin Ma

**Affiliations:** 1 Department of Cardiovascular Medicine, National Clinical Research Center for Geriatric Disorders, Xiangya Hospital, Central South University, No.87 Xiangya Road, Kaifu District, Changsha, 410008 Hunan China; 2 Department of Cardiology, The First Affiliated Hospital of Wenzhou Medical University, Wenzhou, China

**Keywords:** Bisphenols, Non-alcoholic fatty liver disease, Glycolipid, NHANES

## Abstract

**Background:**

Bisphenol A (BPA) and its substitutes bisphenol S (BPS) and bisphenol F (BPF) are endocrine-disrupting chemicals widely used in consumer products, which have been proposed to induce various human diseases. In western countries, one of the most common liver diseases is non-alcoholic fatty liver disease (NAFLD). However, studies on the associations of the three bisphenols with NAFLD in human beings are scarce.

**Methods:**

We included 960 participants aged ≥ 20 years from the NHANES 2013–16 who had available data on levels of urinary BPA, BPS and BPF. The hepatic steatosis index (HSI) > 36 was used to predict NAFLD. Logistic regression analysis and mediation effect analysis were used to evaluate the associations among bisphenols, glycolipid-related markers and NAFLD.

**Results:**

A total of 540 individuals (56.3%) were diagnosed with NAFLD, who had higher concentrations of BPA and BPS but not BPF than those without NAFLD. An increasing trend in NAFLD risks and HSI levels was observed among BPA and BPS tertiles (p for trend < 0.05). After adjustment for confounders, elevated levels of BPA or BPS but not BPF were significantly associated with NAFLD. The odds ratio for NAFLD was 1.581 (95% confidence intervals [CI]: 1.1–2.274, *p* = 0.013) comparing the highest with the lowest tertile of BPA and 1.799 (95%CI: 1.2462.597, *p* = 0.002) for BPS. Mediation effect analysis indicated that serum high-density lipoprotein cholesterol and glucose had a mediating effect on the relationships between bisphenols and NAFLD.

**Conclusions:**

The present study showed that high exposure levels of BPA and BPS increased NAFLD incidence, which might be mediated through regulating glycolipids metabolism. Further studies on the role of bisphenols in NAFLD are warranted.

**Supplementary Information:**

The online version contains supplementary material available at 10.1186/s12940-022-00945-w.

## Introduction

Non-alcoholic fatty liver disease (NAFLD), the most common chronic liver disease, has a global prevalence of approximately 25% among adults and is increasingly recognized as a hepatic manifestation part of metabolic syndrome [1]. Furthermore, the economic burden of NAFLD is projected to increase during the coming decades [2]. Growing evidence indicates that NAFLD frequently coexists with other abnormal conditions and might play a synergistic role in liver injury [3]. Importantly, NAFLD patients commonly tend to have glycolipid metabolism disorders which are shared cardiometabolic risk factors for cardiovascular disease (CVD) [4]. Additionally, NAFLD is obviously related to an increased risk of long-term morbidity and mortality mainly attributable to CVD [5]. Although NAFLD is more prevalent in patients with obesity, insulin resistance, and diabetes, its cause remains largely unknown [2]. In this regard, the importance of chemicals present in consumer products that are widely distributed in the environment is a relatively unexplored factor that could influence NAFLD [6, 7].

Bisphenol A (BPA) is an ingredient of polycarbonate plastics commonly found in food and beverage containers and used as an additive for other plastics [8]. BPA exhibits multiple endocrine-disrupting effects on people of all ages [9]. Considering the substantial potential human health risks based on its endocrine-disrupting feature and toxicological hazards [10, 11], many regulatory agencies have banned the use of BPA [12]. Subsequently, the number of BPA-free products on the market have increased rapidly in the past two decades, and BPA analogues, including bisphenol F (BPF) and bisphenol S (BPS), have gradually been used in some BPA-free products [13, 14]. Consequently, the measured levels of BPS and BPF exposure in human samples markedly rise from 2000 to 2014 [15]. However, the toxicological information of BPF and BPS is limited. Despite an increasing amount of evidence from in vitro and in vivo studies indicated that BPS and BPF, as BPA alternatives, might have similar endocrine-disrupting features as BPA, clinical studies and in vitro and in vivo studies on potential mechanisms for the association of BPS and BPF exposures with human health effects are rare [16].

Therefore, this study was performed to explore the relationships of BPA, BPS and BPF with NAFLD risks in a population-based study from the National Health and Nutrition Examination Survey (NHANES) and to evaluate the underlying role of glycolipid-related markers in these associations.

## Materials and methods

### Study design and participants

NHANES is a nationally cross-sectional survey of the non-institutionalized US residents performed by the National Center for Health Statistics (NCHS) at the US Centers for Disease Control and Prevention (CDC). A stratified, multistage probability cluster design was performed in NHANES and detailed information on demographics, socioeconomic data, personal lifestyle, individual medical and health conditions, and laboratory indicators were collected. The NHANES data are publicly released on a 2-year cycle.

**Fig. 1:**
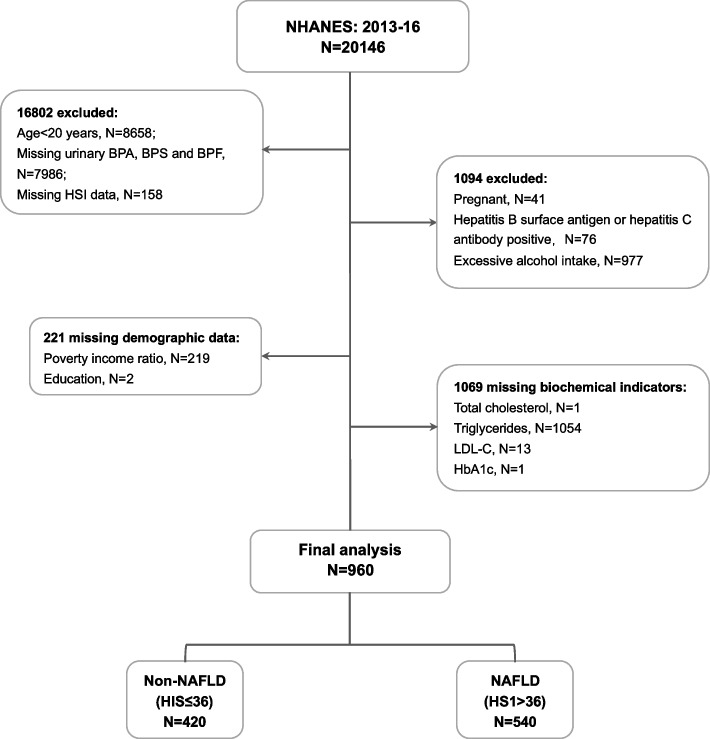
Flowchart of participants for the study. NHANES: National Health and Nutrition Examination Survey; NAFLD: non-alcoholic fatty liver disease; HSI: hepatic steatosis index; BPA: bisphenol A; BPS: bisphenol S; BPF: bisphenol F; BMI: body mass index; LDL-C: low-density lipoprotein cholesterol, HbA1c: glycosylated hemoglobin A1c

The study sample was collected from 2013–14 and 2015–16 continuous cycles of the NHANES given that urinary of BPS and BPF levels were examined in these cycles. The flowchart of this study was shown in Fig. 1. The population in the final analysis consisted of participants aged 20 years and older with complete information, which were used to construct the parameter for calculating the hepatic steatosis index (HSI) to predict NAFLD prevalence, along with data on urinary BPA, BPS, and BPF. Furthermore, subjects who had viral hepatitis (hepatitis B and C) or with excessive alcohol consumption (> 3 drinks/day for males and > 2 drinks/day for females) and pregnant women were excluded. Additionally, we further excluded individuals with missing demographic and biochemical information on key covariates including poverty income ratio, education status, urine creatinine, glycosylated hemoglobin A1c (HbA1c), total cholesterol (TC), triglycerides (TG) and low-density lipoprotein cholesterol (LDL-C). A total of 960 participants were included in the final analysis.

The Institutional Review Board of the NCHS approved this program for NHANES and strictly implemented it in accordance with the Declaration of Helsinki [17]. All participants signed Informed consent forms.

### Measurement of urinary BPA, BPS and BPF concentrations

One-third of random subsamples aged six years or over were selected for determination of urinary bisphenols in NHANES. Urinary levels of BPA, BPS and BPF were measured by online solid-phase extraction coupled with high performance liquid chromatography and tandem mass spectrometry at the Laboratory Science Division of the National Center for Environmental Health of the CDC. This method was described in previous studies [15, 18]. According to the description of the NHANES website, when urinary levels of the three targets were below the lower limits of detection (LLOD), the value obtained by dividing the LLOD by the square root of 2 was routinely substituted (see https://wwwn.cdc.gov/Nchs/Nhanes/2013-2014/EPHPP_H.htm). The LLODs were 0.2 ng/mL for urinary BPA, 0.1 ng/mL for urinary BPS, and 0.2 ng/mL for urinary BPF. In total, 95% of BPA measurements, 55% of BPF measurements, and 90% of BPS measurements were above the LLODs in the current study. Additionally, urinary creatinine was included in all the analysis models of all three targets to correct for urinary dilution in the current study, as done in the previous study [19]. All participants were divided into three subgroups according to tertiles of BPA and BPS, respectively. Because the detection rate of urinary BPF was relatively low, participants for whom the concentration of urinary BPF was not detected categorized as a reference (tertile 1). The remainder of the participants were divided into two groups (tertile 2 and 3) according to the median of urinary BPF value.

### Detection of serum glycolipid-related parameters levels

Blood samples from every participant were collected in the non-fasting state. Serum TG and TC concentrations were detected using specific enzymatic assays. Immunoassays were applied to detect serum high-density lipoprotein cholesterol (HDL-C) concentrations [20]. The values of serum LDL-C were calculated by the Friedewald equation was (LDL-C = TC – HDL-C – TG/5) [21].

Fasting glucose levels were determined by a hexokinase-method (Roche Diagnostics, Indianapolis, IN) [22]. HbA1c levels were quantitatively measured using a Tosoh G8 Glycohemoglobin Analyzer (Tosoh Medics, Inc. San Francisco, CA) [22].

### NAFLD definition

The gold standard for diagnosing NAFLD is liver biopsy, but it is not practical in the general population studies due to the invasiveness and high costs of the procedure. HSI is a common non-invasive screening tool to predict NAFLD presence and is calculated based on the following specific formula = 8 × alanine aminotransferase (ALT, IU/L)/aspartate aminotransferase (AST, IU/L) + body mass index (BMI, kg/m^2^) + 2 (if female) + 2 (if type 2 diabetes). Furthermore, participants were defined as having NAFLD when HSI > 36 according to a previous publication [23].

### Other key covariates

Several potential confounders identified in previous literature [24, 25] were also included in this study as covariates to get closer to reality, including age, sex, ethnicity, family poverty income ratio (< 1, ≥ 1), education status, BMI (continuous), smoking status, drinking status, diabetes and hypertension status. Smoking status was classified into three subgroups according to never smoking before questionnaire (never), quit smoking at least one day (former) and now smoking or have smoked at least one hundred cigarettes (current). Individuals who had two drinks per day for men and one drink per day for women were considered to be drinking. The diagnosis of diabetes and hypertension of participants was based on the clinical criteria or depended on their medical history (self-reported or treated with antihypertensive/antidiabetic drugs) from questionnaires.

### Statistical analysis

Continuous indices were presented as the means (standard deviation) or medians (interquartile range) and categorical indices were expressed as numbers (percentage). The distribution pattern of variables was tested by Kolmogorov–Smirnov analysis. Because TG, BPA, BPS, and BPF levels exhibited skewed distributions, log-transformed levels of the four indicators as continuous variables were applied in our analysis. Student’s t test, analysis of variance, the Mann–Whitney U test, or the λ^2^ test were used to evaluate significant differences in variables between groups where appropriate. Pearson correlation analysis was used to examine the correlations of BPA, BPS and BPF with glycolipid-related parameters and HSI. Logistic regression analysis was applied to examine the odds ratios (ORs) with 95% confidence intervals (CIs) for NAFLD according to tertiles and log-transformed continuous concentrations of urinary BPA, BPS and BPF. Urine creatinine was included in Model 1. Furthermore, ethnicity, education status, drinking, hypertension, diabetes, TG, HDL-C, glucose, HbA1c and urine creatinine were included in model 2. Additionally, model 3 was also adjusted for BPA and/or BPS (log-transformed concentrations) based on model 2 in different conditions. Considering the possible effects of bisphenols on metabolism in participants of different sexes, we performed stratified analyses by sex.

Mediation effect analysis was utilized to evaluate an intermediate variable as a mediator in the pathway between a risk factor and an outcome, estimating the extent to which the effect of the risk factor occurred through the mediator. Mediation effect analysis was performed according to three models [26]: path c for explaining the outcome (Y, NAFLD/HSI) of a risk factor (X, bisphenols) using a logistic regression model (Y is NAFLD) or linear regression model (Y is HSI), path a for explaining the mediator (M, glycolipid-related indicators) for the risk factor using a linear regression model, and path b for estimating the association of the mediator and the risk factor with the outcome using a logistic regression model (Y is NAFLD) or linear regression model (Y is HSI). In addition, mediation effect analysis was performed with adjustments for urine creatinine, ethnicity, education status, hypertension and diabetes (or without diabetes in the mediation effect analysis models of glucose and HbA1c) in the current study. The total effects of bisphenols consisted of the direct effect (DE) and the indirect effect (IE) on NAFLD or HSI. When propositions of a “statistically significant association between bisphenols (X) and mediators (M)” and a “statistically significant association of mediators with NAFLD or HSI (Y)” were true, the intermediate effect occurred as previous description [26].

To examine the robustness of these findings, we performed sensitivity analyses by excluding individuals with levels below the LLOD of urinary BPF because the detection rate of BPF was relatively low. SPSS (version 25.0) and R (version 4.1.3) were used for all statistical analyses in the present study. Here, p < 0.05 was suggested statistically significant difference.

## Results

### Baseline characteristics

In total, 960 adults aged 20 years and older were finally eligible to include in the present study, of whom 444 (46.2%) were male. The mean age of the whole population was 52.9 ± 17.3 years. Among them, 540 individuals, over 50% of the total number, were defined as NAFLD according to HSI > 36. The mean HSI values were 38.5 ± 8.2, 43.9 ± 6.65 and 31.5 ± 3.07 in the total population, the NAFLD and non-NAFLD groups, respectively. The baseline characteristics classified by NAFLD prevalence were presented in Table 1. The interquartile ranges of urinary levels were from 0.5 to 2.2 ng/mL with a median of 1.1 ng/mL for BPA, from 0.2 to 1.2 ng/mL with a median of 0.5 ng/mL for BPS, and from 0.14 to 0.8 ng/mL with a median of 0.25 ng/mL for BPF. Participants with NAFLD were more prone to be non-Hispanic black (25.7%), received less than a high school education (25%), and suffering from hypertension (54.8%) and diabetes (34.1%), and were prevalent in higher BMI, TG, glucose and HbA1c levels and lower of HDL-C levels compared to those without NAFLD (all *p* < 0.05). Moreover, individuals with NAFLD had elevated urinary concentrations of BPA and BPS compared with those without NAFLD [BPA: 1.25 (0.6, 2.32) vs 0.9 (0.5, 2) ng/mL, *p* = 0.001; BPS: 0.6 (0.2, 1.33) vs 0.4 (0.2, 0.95) ng/mL, *p* < 0.001]. However, no significant differences with regard to age, sex, family poverty income ratio, smoking status, TC, LDL-C and BPF levels were noted between the NAFLD and non-NAFLD groups (all *p* > 0.05). The demographic characteristics were similar between individuals with NAFLD and without NAFLD in females and males with the exception of education status (shown in Supplementary Table S1). Moreover, elevated urinary concentrations of BPA and BPS but not BPF were observed in NAFLD group compared with non-NAFLD groups in both females and males (Supplementary Table S1). These findings were consistent with the analysis results for the total population. Additionally, only urinary BPA levels were higher in females than males among the three bisphenols [BPA: 1.15 (0.5, 2.6) vs 1(0.5, 2) ng/mL, *p* = 0.025, Supplementary Table S2).

**Table 1:** Baseline characteristics of the study population according to NAFLD and non-NAFLD, NHANES 2013–16

**Variables**		**Total**		
	**Overall**	**Non-NAFLD**	**NAFLD**	
	***N* = *960***	***N* = *420***	***N* = *540***	***p* value**
Age ^a^	52.9 (17.3)	53.1 (18.5)	52.8 (16.3)	0.833
Sex, n(%)				0.063
Female	516 (53.8%)	211 (50.4%)	305 (56.5%)	
Male	444 (46.2%)	209 (49.8%)	235 (43.5%)	
Ethnicity, n(%)				< 0.001
Non-Hispanic White	367 (38.2%)	169 (40.2%)	198 (36.7%)	
Non-Hispanic Black	212 (22.1%)	73 (17.4%)	139 (25.7%)	
Non-Hispanic Asian	129 (13.4%)	96 (22.9%)	33 (6.11%)	
Mexican American	130 (13.6%)	36 (8.57%)	94 (17.4%)	
Other Hispanic	122 (12.7%)	46 (11.0%)	76 (14.1%)	
Education, n(%)				0.001
Less than high school	210 (21.9%)	75 (17.9%)	135 (25.0%)	
High school or equivalent	210 (21.9%)	80 (19.0%)	130 (24.1%)	
College or above	540 (56.2%)	265 (63.1%)	275 (50.9%)	
Poverty income ratio				0.117
< 1.0	209 (21.8%)	81 (19.3%)	128 (23.7%)	
≥ 1.0	751 (78.2%)	339 (80.7%)	412 (76.3%)	
Smoking, n(%)				0.906
never	596 (62.1%)	261 (62.3%)	335 (62.0%)	
former	229 (23.9%)	98 (23.3%)	131 (24.3%)	
current	135 (14.1%)	61 (14.5%)	74 (13.7%)	
Drinking, n(%)				0.345
No or unknown	293 (30.6%)	121 (28.8%)	172 (31.9%)	
Yes	667 (69.5%)	299 (71.2%)	368 (68.1%)	
Hypertension, n(%)				< 0.001
No	504 (52.5%)	260 (61.9%)	244 (45.2%)	
Yes	456 (47.5%)	160 (38.1%)	296 (54.8%)	
Diabetes, n(%)				< 0.001
No	722 (75.2%)	366 (87.1%)	356 (65.9%)	
Yes	238 (24.8%)	54 (12.9%)	184 (34.1%)	
BMI, (kg/m^2^) ^a^	29.3 (7.08)	23.8 (2.92)	33.6 (6.35)	< 0.001
TC, (mg/dL) ^a^	188 (39.7)	188 (39.4)	188 (39.9)	0.910
TG, (mg/dL) ^b^	94.0 [63.0;136]	80.0 [56.0;112]	108 [74.0;155]	< 0.001
LDL-C, (mg/dL) ^a^	112 (34.8)	109 (33.7)	113 (35.7)	0.083
HDL-C, (mg/dL) ^a^	54.6 (16.1)	60.1 (17.5)	50.4 (13.5)	< 0.001
Glucose, (mg/dL) ^a^	111 (37.4)	102 (25.6)	119 (43.1)	< 0.001
HbA1c (%) ^a^	5.92 (1.26)	5.60 (0.87)	6.16 (1.45)	< 0.001
Urinary BPA, (ng/mL) ^b^	1.10 [0.50;2.20]	0.90 [0.50;2.00]	1.25 [0.60;2.32]	0.001
Urinary BPS, (ng/mL) ^b^	0.50 [0.20;1.20]	0.40 [0.20;0.95]	0.60 [0.20;1.33]	< 0.001
Urinary BPF, (ng/mL) ^b^	0.25 [0.14;0.80]	0.20 [0.14;0.70]	0.30 [0.14;0.80]	0.231
HSI ^a^	38.5 (8.20)	31.5 (3.07)	43.9 (6.65)	< 0.001

^a^ data is expressed with mean (SD)

^b^ data is expressed with median [IQR]. *NHANES* National Health and Nutrition Examination Survey, *NAFLD* non-alcoholic fatty liver disease, *SD* standard deviation, *IQR* interquartile range, *BMI* body mass index, *TC* total cholesterol, *TG* triglycerides, *LDL-C* low-density lipoprotein cholesterol, *HDL-C* high-density lipoprotein cholesterol, *HbA1c* glycosylated hemoglobin A1c, *BPA* bisphenol A, *BPS* bisphenol S, *BPF* bisphenol F, *HSI* hepatic steatosis index, *p* < 0.05 suggests significant differences

Additionally, as shown in Table 2, we divided the whole population into three subgroups according to the tertile concentrations of urinary BPA and BPS and the levels of BPF. Participants with the highest tertile of BPA were more likely to be non-Hispanic white, have elevated levels of BMI, BPS and BPF, and have lower levels of HDL-C than those with the lowest tertile of BPA (all *p* < 0.05). Younger non-Hispanic blacks and subjects with a lower poverty income ratio and higher BMI, glucose, HbA1c and BPA levels tended to have elevated urinary concentrations of BPS (all *p* < 0.05). The following features were noted in high BPF levels group: non-Hispanic Whites, current smokers and drinkers, higher levels of education, higher BMI, BPA and BPS levels, and lower concentrations of TC and LDL-C were prevalent in the high level of urinary BPF group (all *p* < 0.05). Of note, ascending trends in NAFLD incident rates and HSI levels were observed in individuals who had higher urinary levels of BPA and BPS (all *p* < 0.05). However, NAFLD incidence and HSI levels were not significantly different among BPF tertiles.

**Table 2:** Baseline characteristics of the study population by urinary BPA, BPF, and BPS levels tertiles, NHANES 2013–16

	**Urinary BPA levels**			**Urinary BPS levels**			**Urinary BPF levels**		
	**Tertile 1**	**Tertile 2**	**Tertile 3**	**Tertile 1**	**Tertile 2**	**Tertile 3**	**Tertile 1**	**Tertile 2**	**Tertile 3**
**Variables**	***N* = *301***	***N* = *325***	***N* = *334***	***N* = *291***	***N* = *327***	***N* = *342***	***N* = *435***	***N* = *284***	***N* = *241***
Age ^a†^	54.2 (16.5)	52.2 (17.4)	52.4 (17.8)	54.8 (17.4)	52.8 (16.9)	51.4 (17.4)	52.3 (17.2)	53.9 (17.0)	52.6 (17.7)
Sex, n(%)									
Female	172 (57.1%)	182 (56.0%)	162 (48.5%)	150 (51.5%)	181 (55.4%)	185 (54.1%)	240 (55.2%)	146 (51.4%)	130 (53.9%)
Male	129 (42.9%)	143 (44.0%)	172 (51.5%)	141 (48.5%)	146 (44.6%)	157 (45.9%)	195 (44.8%)	138 (48.6%)	111 (46.1%)
Ethnicity, n(%) ^*†!^									
Non-Hispanic White	103 (34.2%)	124 (38.2%)	140 (41.9%)	134 (46.0%)	127 (38.8%)	106 (31.0%)	132 (30.3%)	114 (40.1%)	121 (50.2%)
Non-Hispanic Black	43 (14.3%)	76 (23.4%)	93 (27.8%)	39 (13.4%)	73 (22.3%)	100 (29.2%)	80 (18.4%)	59 (20.8%)	73 (30.3%)
Non-Hispanic Asian	73 (24.3%)	37 (11.4%)	19 (5.69%)	58 (19.9%)	44 (13.5%)	27 (7.89%)	89 (20.5%)	31 (10.9%)	9 (3.73%)
Mexican American	48 (15.9%)	42 (12.9%)	40 (12.0%)	36 (12.4%)	35 (10.7%)	59 (17.3%)	80 (18.4%)	34 (12.0%)	16 (6.64%)
Other Hispanic	34 (11.3%)	46 (14.2%)	42 (12.6%)	24 (8.25%)	48 (14.7%)	50 (14.6%)	54 (12.4%)	46 (16.2%)	22 (9.13%)
Education, n(%)									
Less than high school	61 (20.3%)	84 (25.8%)	65 (19.5%)	59 (20.3%)	71 (21.7%)	80 (23.4%)	103 (23.7%)	64 (22.5%)	43 (17.8%)
High school or equivalent	66 (21.9%)	63 (19.4%)	81 (24.3%)	51 (17.5%)	80 (24.5%)	79 (23.1%)	100 (23.0%)	54 (19.0%)	56 (23.2%)
College or above	174 (57.8%)	178 (54.8%)	188 (56.3%)	181 (62.2%)	176 (53.8%)	183 (53.5%)	232 (53.3%)	166 (58.5%)	142 (58.9%)
Poverty income ratio†									
< 1.0	61 (20.3%)	66 (20.3%)	82 (24.6%)	47 (16.2%)	73 (22.3%)	89 (26.0%)	98 (22.5%)	59 (20.8%)	52 (21.6%)
≥ 1.0	240 (79.7%)	259 (79.7%)	252 (75.4%)	244 (83.8%)	254 (77.7%)	253 (74.0%)	337 (77.5%)	225 (79.2%)	189 (78.4%)
Smoking, n(%)									
never	193 (64.1%)	206 (63.4%)	197 (59.0%)	183 (62.9%)	194 (59.3%)	219 (64.0%)	290 (66.7%)	174 (61.3%)	132 (54.8%)
former	69 (22.9%)	74 (22.8%)	86 (25.7%)	73 (25.1%)	82 (25.1%)	74 (21.6%)	91 (20.9%)	70 (24.6%)	68 (28.2%)
current	39 (13.0%)	45 (13.8%)	51 (15.3%)	35 (12.0%)	51 (15.6%)	49 (14.3%)	54 (12.4%)	40 (14.1%)	41 (17.0%)
Drinking, n(%)^!^									
No or unknown	99 (32.9%)	102 (31.4%)	92 (27.5%)	91 (31.3%)	90 (27.5%)	112 (32.7%)	154 (35.4%)	69 (24.3%)	70 (29.0%)
Yes	202 (67.1%)	223 (68.6%)	242 (72.5%)	200 (68.7%)	237 (72.5%)	230 (67.3%)	281 (64.6%)	215 (75.7%)	171 (71.0%)
Hypertension, n(%)									
No	155 (51.5%)	183 (56.3%)	166 (49.7%)	158 (54.3%)	168 (51.4%)	178 (52.0%)	233 (53.6%)	143 (50.4%)	128 (53.1%)
Yes	146 (48.5%)	142 (43.7%)	168 (50.3%)	133 (45.7%)	159 (48.6%)	164 (48.0%)	202 (46.4%)	141 (49.6%)	113 (46.9%)
Diabetes, n(%)									
No	223 (74.1%)	252 (77.5%)	247 (74.0%)	225 (77.3%)	249 (76.1%)	248 (72.5%)	319 (73.3%)	224 (78.9%)	179 (74.3%)
Yes	78 (25.9%)	73 (22.5%)	87 (26.0%)	66 (22.7%)	78 (23.9%)	94 (27.5%)	116 (26.7%)	60 (21.1%)	62 (25.7%)
BMI, (kg/m^2^) ^a *†!^	28.5 (6.80)	28.7 (6.43)	30.7 (7.73)	28.2 (6.91)	29.1 (6.69)	30.6 (7.42)	28.9 (6.85)	29.4 (7.01)	30.0 (7.54)
TC, (mg/dL) ^a !^	192 (40.8)	188 (38.3)	184 (39.8)	190 (39.8)	187 (41.4)	186 (37.8)	192 (41.8)	188 (37.6)	181 (37.1)
TG, (mg/dL) ^b *^	95.0 [65.0;146]	87.0 [58.0;123]	100 [65.2;139]	94.0 [65.0;136]	91.0 [64.0;134]	99.0 [61.0;138]	96.0 [65.0;133]	92.5 [63.0;139]	91.0 [58.0;135]
LDL-C, (mg/dL) ^a!^	114 (36.1)	112 (34.1)	109 (34.3)	113 (34.0)	112 (37.4)	111 (33.0)	115 (36.9)	111 (33.6)	107 (31.8)
HDL-C, (mg/dL) ^a *^	55.4 (15.2)	55.9 (16.8)	52.6 (15.9)	55.1 (15.2)	54.6 (15.7)	54.2 (17.2)	55.2 (16.2)	54.5 (16.9)	53.6 (14.7)
Glucose, (mg/dL) ^a†^	111 (36.2)	112 (42.3)	111 (33.3)	108 (30.2)	110 (35.8)	116 (43.6)	114 (42.4)	109 (29.4)	110 (36.0)
HbA1c (%) ^a†^	5.91 (1.26)	5.97 (1.40)	5.87 (1.10)	5.82 (1.07)	5.87 (1.17)	6.04 (1.46)	6.00 (1.41)	5.85 (1.10)	5.84 (1.13)
Urinary BPA, (ng/mL)^b *†!^	0.40 [0.20;0.50]	1.10 [0.80;1.30]	3.00 [2.10;4.68]	0.70 [0.40;1.40]	1.20 [0.60;2.40]	1.40 [0.70;2.80]	0.80 [0.40;1.70]	1.30 [0.60;2.40]	1.40 [0.70;3.40]
Urinary BPS, (ng/mL)^b *†^!	0.30 [0.10;0.90]	0.50 [0.20;1.10]	0.70 [0.30;1.60]	0.10 [0.07;0.20]	0.50 [0.30;0.60]	1.60 [1.10;3.00]	0.50 [0.20;1.10]	0.50 [0.20;1.12]	0.60 [0.30;1.40]
Urinary BPF, (ng/mL) ^b *!^	0.14 [0.14;0.50]	0.20 [0.14;0.70]	0.40 [0.14;1.10]	0.20 [0.14;0.60]	0.30 [0.14;0.90]	0.30 [0.14;0.90]	0.14 [0.14;0.14]	0.40 [0.30;0.50]	1.90 [1.10;5.30]
HSI ^a *†^	37.7 (7.95)	37.9 (7.72)	39.8 (8.71)	37.2 (7.86)	38.2 (7.84)	39.9 (8.61)	38.2 (8.11)	38.4 (7.93)	39.2 (8.65)
NAFLD, n(%) ^*†^									
No	150 (49.8%)	145 (44.6%)	125 (37.4%)	152 (52.2%)	147 (45.0%)	121 (35.4%)	196 (45.1%)	125 (44.0%)	99 (41.1%)
Yes	151 (50.2%)	180 (55.4%)	209 (62.6%)	139 (47.8%)	180 (55.0%)	221 (64.6%)	239 (54.9%)	159 (56.0%)	142 (58.9%)

^a^ data is expressed with mean (SD)

^b^ data is expressed with median [IQR]. *NHANES* National Health and Nutrition Examination Survey, *NAFLD* non-alcoholic fatty liver disease, *SD* standard deviation, *IQR* interquartile range, *BMI* body mass index, *TC* total cholesterol, *TG* triglycerides, *LDL-C* low-density lipoprotein cholesterol, *HDL-C* high-density lipoprotein cholesterol, *HbA1c* glycosylated hemoglobin A1c, *BPA* bisphenol A, *BPS* bisphenol S, *BPF* bisphenol F, *HSI* hepatic steatosis index

^*^Significant difference across tertiles for BPA

^†^Significant difference across tertiles for BPS

^!^Significant difference across tertiles for BPF, *p* < 0.05 suggests significant differences

### Associations of urinary bisphenols with NAFLD

Logistic regression models were applied to analyze the associations between the three bisphenols and NAFLD risks, as summarized in Table 3. When only adjusting for urine creatine levels in model 1, elevated urinary levels of BPA and BPS, but not BPF, were significantly related to the risk of NAFLD (all *p* < 0.05). Because BMI was the one of the key parts of the special equation to calculate the HSI, it was not possible to adjust BMI in multivariate analysis for the NAFLD prediction model based on the HSI, as previously recommended [27]. In model 2, some key covariates including ethnicity, education status, drinking status, hypertension, diabetes, TG, HDL-C, glucose and HbA1c, were adjusted based on model 1. After adjustment for these covariates in model 2, comparing the highest with lowest tertile of bisphenols, the OR with 95%CI for NAFLD was 1.786 (1.255–2.542, *p* = 0.001) for BPA, 1.97 (1.381–2.811, *p* < 0.001) for BPS and 1.344 (0.937–1.929, *p* = 0.108) for BPF. In model 3, urinary of BPA and/or BPS levels (as log-transformation) were further mutually adjusted under different conditions based on model 2. Participants in the highest tertile of BPA and BPS had 58.1% (OR: 1.581, 95%CI: 1.1–2.274) and 79.9% (OR: 1.799, 95%CI: 1.246–2.597) elevated risks for NAFLD, respectively, compared to those in the lowest tertile subgroups. As continuous variables, per log unit increases in levels of BPA and BPS were significantly related to 1.391-fold (95%CI: 1.007–1.921) and 1.476-fold (95%CI: 1.139–1.913) risks for NAFLD, respectively, after adjusting for all multiple confounding factors. No significant relationship was observed between BPF and NAFLD regardless of whether BPF was included as a categorical or continuous variable in logistic regression models.

**Table 3:** Associations of urinary BPA, BPS, and BPF levels with NAFLD

	**Tertile 1**	**Tertile 2**		**Tertile 3**		**Per Log unit increase**	
		**OR(95%CI)**	**p**	**OR(95%CI)**	**p**	**OR(95%CI)**	**p**
**BPA**							
Model 1	1 (ref)	1.25(0.912,1.714)	0.166	1.693(1.231,2.327)	0.001	1.531(1.158,2.025)	0.003
Model 2	1 (ref)	1.49(1.045,2.125)	0.028	1.786(1.255,2.542)	0.001	1.568(1.147,2.144)	0.005
Model 3	1 (ref)	1.43(0.999,2.047)	0.051	1.581(1.100,2.274)	0.013	1.391(1.007,1.921)	0.045
**BPS**							
Model 1	1 (ref)	1.339(0.975,1.838)	0.071	1.988(1.444,2.738)	< 0.001	1.573(1.253,1.974)	< 0.001
Model 2	1 (ref)	1.346(0.948,1.909)	0.096	1.970(1.381,2.811)	< 0.001	1.576(1.225,2.026)	< 0.001
Model 3	1 (ref)	1.253(0.876,1.792)	0.216	1.799(1.246,2.597)	0.002	1.476(1.139,1.913)	0.003
**BPF**							
Model 1	1 (ref)	1.054(0.779,1.425)	0.733	1.200(0.871,1.655)	0.265	1.227(0.976,1.541)	0.079
Model 2	1 (ref)	1.131(0.808,1.582)	0.473	1.344(0.937,1.929)	0.108	1.280(0.989,1.656)	0.061
Model 3	1 (ref)	1.056(0.749,1.489)	0.755	1.192(0.823,1.726)	0.353	1.165(0.895,1.517)	0.256

Model 1 is adjusted urine creatinine (tertiles); Model 2 is adjusted for ethnicity (non-Hispanic white, non-Hispanic black, non-Hispanic Asian, Mexican American, Other Hispanic), education (less than high school, high school or equivalent, college or above), drinking (no or unknown, yes), hypertension (no, yes), diabetes (no, yes), log-transformed levels of triglyceride, high-density lipoprotein cholesterol, glucose, glycosylated hemoglobin A1c and urine creatinine (tertiles), Model 3 is adjusted variables in Model 2 plus log-transformed concentration of BPA and/or BPS. *BPA* bisphenol A, *BPS* bisphenol S, *BPF* bisphenol F, *OR* odds ratio, *CI* confidence intervals, *p* < 0.05 suggests significant differences

Furthermore, the gender stratification results of associations between three bisphenols and NAFLD risks were presented in Supplementary Table S3. There was no significant interaction between sex and three bisphenols levels (all p for interaction > 0.05), although the relationship between BPS and NAFLD seemed stronger in females than males. Comparing the highest with the lowest tertile of urinary BPS, the OR for NAFLD was 2.304 (95%CI:1.394–3.807, *p* = 0.002 for trend) in females and 1.623 (95%CI: 0.979–2.691, *p* = 0.054 for trend) in males after adjusting for multiple confounders. The associations of BPF and BPA with NAFLD did not differ by sex (Supplementary Table S3). In multivariable adjusted models, no significant effect of urinary BPF levels on NAFLD and an obvious effect of urinary BPA levels on NAFLD were observed both in women and men (Supplementary Table S3), which were consistent with that obtained for the total population.

Additionally, as shown in Supplementary Table S4, the associations of urinary bisphenols levels except BPA with NAFLD did not change appreciably in sensitivity analyses when excluding individuals with urinary BPF levels below the LLOD.

**Fig. 2:**
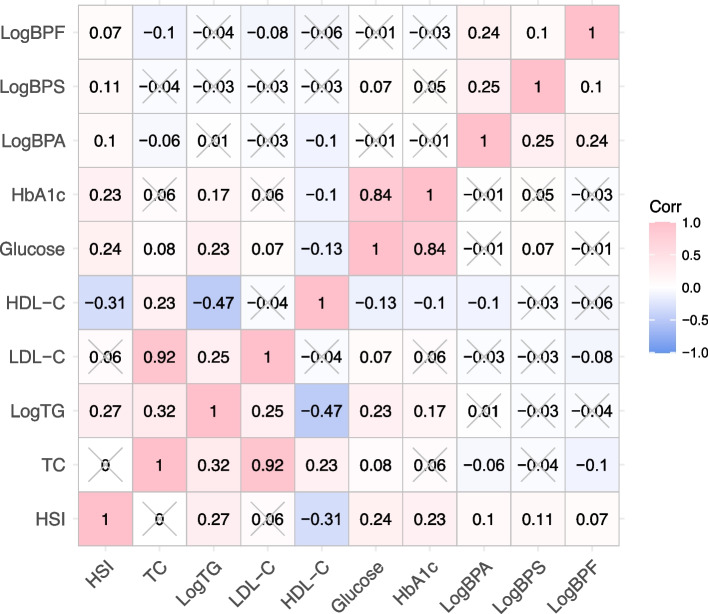
Correlations of urinary BPA, BPS, and BPF levels with HSI and glycolipid-related indicators. TC: total cholesterol; TG: triglycerides; LDL-C: low- density lipoprotein cholesterol; HDL-C: high-density lipoprotein cholesterol; HbA1c: glycosylated hemoglobin A1c; BPA: bisphenol A; BPS: bisphenol S; BPF: bisphenol F; HSI: hepatic steatosis index, *p* < 0.05 suggests significant differences. ✕ suggests no significant difference

Equally important, Pearson correlation analysis showed that log-transformed urinary BPA and BPS levels were significantly and positively correlated with HSI (r = 0.1 between BPA and HSI, r = 0.11 between BPS and HSI, and r = 0.07 between BPF and HSI, all *p* < 0.05), as presented in Fig. 2.

### Roles of glycolipid-related indices in the relationships of urinary bisphenols with NAFLD

**Fig. 3:**
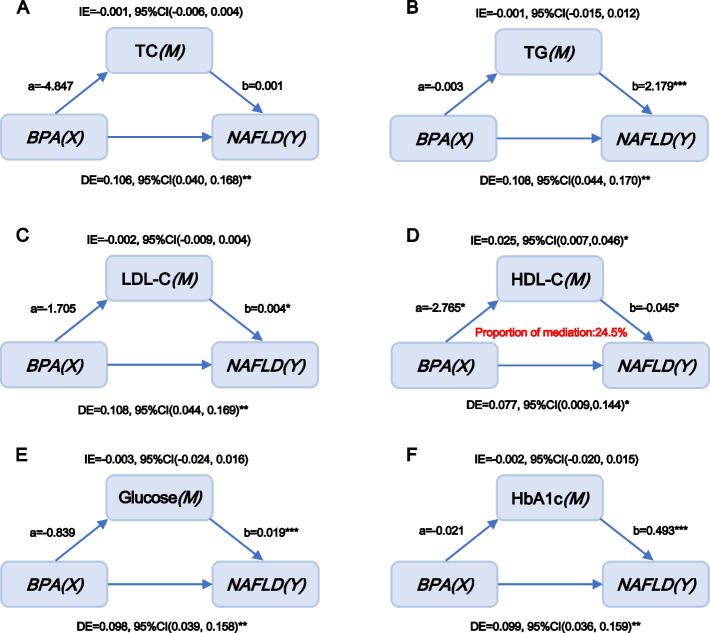
Mediation effect analysis of serum glycolipid-related indicators on the interaction between urinary BPA and NAFLD. NAFLD: non-alcoholic fatty liver disease; TC: total cholesterol; TG: triglycerides; LDL-C: low-density lipoprotein cholesterol; HDL-C: high-density lipoprotein cholesterol; HbA1c: glycosylated hemoglobin A1c; BPA: bisphenol A; IE: indirect effect; DE: direct effect, * *p* < 0.05, ** *p* < 0.01, *** *p* < 0.001. *p* < 0.05 suggests significant differences

**Fig. 4:**
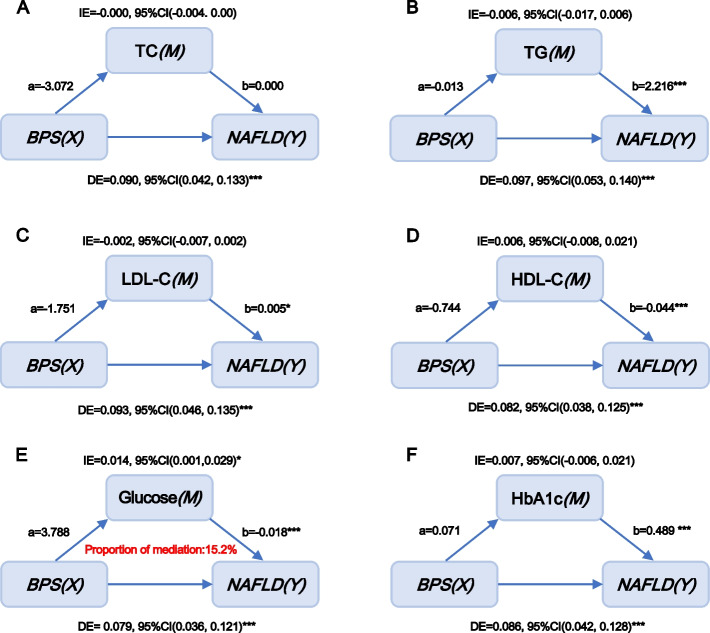
Mediation effect analysis of serum glycolipid-related indicators on the interaction between urinary BPS and NAFLD. NAFLD: non-alcoholic fatty liver disease; TC: total cholesterol; TG: triglycerides; LDL-C: low-density lipoprotein cholesterol; HDL-C: high-density lipoprotein cholesterol; HbA1c: glycosylated hemoglobin A1c; BPS: bisphenol S; IE: indirect effect; DE: direct effect, * *p* < 0.05, ** *p* < 0.01, *** *p* < 0.001. *p* < 0.05 suggests significant differences

**Fig. 5:**
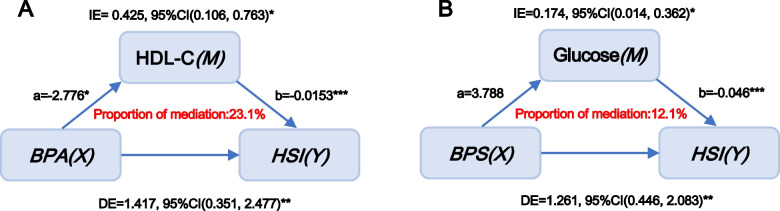
Mediation effect analysis of serum HDL-C or glucose on the interaction between urinary BPA or BPS and HSI. HDL-C: high-density lipoprotein cholesterol; HSI: hepatic steatosis index; BPA: bisphenol A; BPS: bisphenol S; IE: indirect effect; DE: direct effect, * *p* < 0.05, ** *p* < 0.01, *** *p* < 0.001. *p* < 0.05 suggests significant differences

According to Pearson correlation analysis, the correlation coefficients among urinary bisphenols, serum glucolipid-related indices and HSI were presented in Fig. 2. A significantly negative correlation of BPA with HDL (r = -0.1, *p* < 0.05) and a significantly positive association of BPS with glucose (r = 0.07, *p* < 0.05) were found in the current study. Furthermore, HSI levels were remarkably and positively correlated with TG, glucose and HbA1c levels and negatively correlated with HDL-C levels in the entire population (r = 0.27 for TG, r = 0.24 for glucose, r = 0.23 for HbA1c and r = -0.31 for HDL-C, all *p* < 0.05, Fig. 2). Because no significant association between BPF and NAFLD/HSI was observed, mediation effect analysis was performed to identify whether glucolipid-related indicators mediated the efects of BPA and BPS on NAFLD/HSI. The role of glucolipid-related indicators levels in the associations of bisphenols with NAFLD was explored in a model with NAFLD/HSI as the dependent variable, urinary bisphenols levels as the independent variable, and glucolipid-related indicators levels as the mediator variable, and urine creatinine, ethnicity, education status, hypertension and diabetes (or without diabetes where appropriate) as covariates. Interestingly, the results presented in Figs. 3 and 4 revealed that bisphenols may participate in the development of NAFLD by mediating serum HDL-C and glucose levels. Specifically, the mediated efficacy of HDL-C accounted for 24.5% in the relationship between BPA and NAFLD development (IE = 0.025, 95%CI: 0.007–0.046, *p* < 0.05, Fig. 3), whereas the mediated efficacy of glucose accounted for 15.2% of the predictive ability of BPS on NAFLD (IE = 0.014, 95%CI: 0.001–0.029, *p* < 0.05, Fig. 4). In addition, we further analyzed the mediated effects of glucose-mediated indices on the relationships of BPA and BPS with HSI (Fig. 5). Similarly, HDL-C and glucose had significant mediation effects on the associations between bisphenols and HSI. The estimate of IE between BPA and HSI through HDL-C was 0.425 (95%CI: 0.106–0.763, *p* < 0.05), with a 23.1% of mediated efficacy. Moreover, the mediation efficacy of glucose accounted for 12.1% of the relation between BPS and HSI (IE = 0.174, 95%CI: 0.014–0.362, *p* < 0.05).

## Discussion

This study explored the associations of multiple bisphenols including BPA, BPS and BPF with NAFLD prevalence and first investigated the role of glycolipid-related factors in these relationships. In this US population-based study, our results revealed that adults with elevated concentrations of urinary BPA and BPS, but not BPF, tended to develop NAFLD after adjusting for relevant confounders, including sociodemographic variables, lifestyle factors, clinical and laboratory parameters. More importantly, mediation effect analysis indicated that serum glycolipid-related indices, such as HDL-C and glucose, potentially exhibit mediated effects on the associations of bisphenols with the prevalence of NAFLD. These findings further provided critical information with regard to the potential relations between bisphenols and NAFLD and insights into the underlying mechanisms of bisphenols in NAFLD development.

In the past decades, BPA had been used in various industrial applications, especially for the production of polycarbonate and plastic epoxy [8]. Currently, BPA has become one of the highest produced volumes among chemicals worldwide [8]. Given the numerous applications of BPA, people have expressed concerns regarding whether it is harmful to human health. The wealth of studies have proven toxicological properties and endocrine-disrupting characteristics of BPA on human diseases, such as CVD and metabolic disorders [28, 29]. When BPA was restricted in numerous consumer products since 2016, other bisphenols to replace BPA were emerging in industrial production [12]. Subsequently, BPS and BPF are considered suitable substitutes for BPA and are widely used in industrial applications [12]. Although BPS and BPF are thought to be endocrine-disrupting and toxic chemicals given their similar chemical structures to BPA [16], less information is available regarding the role of BPS and BPF in adverse human health conditions. Notably, human exposure to these three bisphenols is widespread by a variety of ways in the general population [12], so biomonitoring of these chemicals and studying on their relationships and mechanisms in human diseases are a real concern.

NAFLD has an estimated prevalence of 25% in adults worldwide, with a continuously increasing trend [30, 31]. Unfortunately, during the coming decades, the economic burden of NAFLD will likely increase rapidly due to the high disease prevalence and complications [31, 32]; thus, its underlying influencing factors should be identified. Emerging evidence has revealed the detrimental role of environmental toxins in liver injury, particularly in the incidence and progression of NAFLD [33]. Exposure to bisphenols, especially BPA, as a ubiquitous endocrine-disrupting and toxic chemical, has also been reported to be associated with obesity, diabetes, and CVD [24, 29, 34]. In addition, data on the effects of BPA on NAFLD are increasing [27, 35]. Several cross-sectional studies revealed that urinary concentrations of BPA were significantly related to NAFLD in Hispanic adolescents and US adults [27, 35]. However, population-based studies exploring the relationships of BPS and BPF with NAFLD are scarce. In the present study, our findings also supported previous results of a positive association of urinary BPA concentrations and NAFLD prevalence [27, 35]. Moreover, our study first investigated the relationships of BPA alternatives, including BPS and BPF, with NAFLD risks, and a significant and positive relationship with NAFLD was observed for BPS but not BPF in this cohort of 960 adults, further providing more information on the contribution of bisphenols to the occurrence of NAFLD. In addition, we found a stronger association between BPS and NAFLD in women than men, although there was no difference in urinary BPS concentration between men and women in the current study. The estrogenic activities of BPS may explain such phenomenon but the underlying mechanisms remain to be understood.

Of note, pathogenic pathways of NAFLD are influenced by multiple metabolic factors, including abnormal glucose metabolism (due to insulin resistance) and lipid abnormalities (for example, low HDL-C and high TG) [2]. Moreover, given its endocrine-disrupting characteristic, BPA is thought to participate in de novo fatty acid synthesis [36], stimulate the accumulation of TG in adipocytes and human hepatocellular carcinoma cells [37] and up-regulate the expression of genes involved in lipid metabolism [36]. Similar to BPA, BPF and BPS are considered as endocrine-disrupting chemicals and have some BPA like estrogenic activities [16]. Furthermore, it has been proposed that BPS and BPF enhance lipid accumulation and promote the differentiation of preadipocytes into adipocytes through the PPARγ signaling pathway [38–40]. Additionally, the exposure to BPA and BPS resulted in the differential expression of genes related to lipid metabolism and adipogenesis and also increasing 3T3-L1 adipocyte differentiation [41–43]. Notably, BPA can disturb glucose homeostasis and influence the pancreatic β-cell function through multiple mechanisms [44]. BPS also plays critical roles in disturbance of glucose metabolism [45]. Although dysglycemia and lipid abnormalities appear to bridge the link between bisphenols and NAFLD based on the above data, whether glycolipid metabolism disorders affect the relationship between bisphenols and the occurrence and development of NAFLD remains unknown. Therefore, the current study first investigated the potential role of serum glycolipid-related parameters in the association of bisphenols and NAFLD using mediation effect analyses. Interestingly, a significant mediated efficacy for HDL-C in the relation of BPA with NAFLD and for serum glucose in the relation of BPS with NAFLD were found, indicating abnormal glucolipid metabolism may mediate the associations between bisphenols NAFLD. Further studies are needed to confirm these findings and assess possible glucolipid metabolism mechanisms explaining the effects of bisphenols on NAFLD.

Due to the relatively high percentage of participants with urinary BPF levels below the LLOD, we did sensitivity analyses in those with BPF levels above the LLOD and found that the significant association of BPA with NAFLD disappeared. Here, the relatively small sample size of sensitivity analyses (*N* = 525) might explain this result. Regarding the negative result between BPF and NAFLD, we had to consider the influence of that the relatively high percentage of participants with urinary BPF levels below the LLOD, and the exposure levels of BPF were approximately one-fifth of BPA levels and one-half of BPS levels in the whole population. Therefore, although we did not observe a positive association between BPF and NAFLD in the current study, attention should still be given to BPF exposure levels and its harmful effects on human health. Future prospective cohort studies including participants exposed to high concentrations of urinary BPF need to be conducted.

This study analyzed a nationally representative data from the NHANES study, which can avoid the effect on the generalizability of the results and enable our findings to be generalized to a broader population. In addition, we, for the first time, observed the relationships among three target bisphenols, NAFLD and serum glucolipid-related indicators in a population.

Nevertheless, several limitations should be noted in this study. First, given the inherent features of the cross-sectional and observational study, only the relationship between bisphenols and NAFLD as well as glycolipid-related parameters, could be clarified, the causal inference could not be assessed. Second, in the current study, after exclusion of other causes of chronic liver disease, the non-invasive index (HSI) was used to define NAFLD, which can inevitably lead to misdiagnosis of the presence or absence of NAFLD. Even though the diagnostic golden standard for NAFLD is liver biopsy, it is not applicable in large population-based studies. Moreover, the HSI, which includes important liver enzyme indicators (ALT and AST), has been recognized as a reliable non-invasive diagnostic method for NAFLD in populations [46]. The exact correlation between bisphenols exposure and the development of NAFLD should be demonstrated in the future. Third, although we included as many confounding factors as possible based on NHANES data, the potential influences of unmeasured confounders on our study cannot be ruled out. Fourth, the proportion of subjects with urinary BPF concentrations below the LLOD was relatively high. Although this did not appreciably affect the categorization of BPF exposure in our categorical data analysis, we further performed sensitivity analyses in the population with concentrations of urinary BPF above the LLOD. Fifth, NHANES staff collected spot urine samples of participants to detect levels of bisphenols, which could bring a considerable intra-individual and inter-person temporal variability of urinary bisphenols levels. However, the population from the NHANES study is sufficiently large and urine samples of every participant are randomly collected relative to eating time and bladder-emptying time, therefore, the urinary bisphenols concentration based on the analysis of one spot urine sample can fully reflect the average exposure of the population to bisphenols as noted in a previous study [47]. Finally, information about sources of BPA, BPS and BPF is limited in NHANES, and individual confounding patterns of the three exposures could not be determined. Further studies need to confirm these associations.

## Conclusions

In this population-based, cross-sectional study, we found that urinary BPA and BPS levels were significantly associated with NAFLD risks and that serum HDL-C and glucose had obvious mediation effects on these relationships, suggesting that biomonitoring and avoiding daily exposure to bisphenols represent possible strategies for the prevention of NAFLD.

## Supplementary Information

**Additional file 1: Table S1. **Baseline characteristicsof the study population according to NAFLD and non-NAFLD stratified by sex, NHANES2013–16. **Table S2. **Urinary concentrations of BPA, BPF and BPS according to sex. **Table S3. **Associations of urinary BPA, BPS, and BPF levels with NAFLD stratified by sex. **Table S4.** Associations of urinary BPA, BPS, and BPF levels with NAFLD in participants with urinary BPF concentrations above the LLOD (*N*=525).

## Data Availability

The data for this study were obtained from the public database of the National Center for Health Statistics. https:// www.cdc.gov/nchs/nhanes/.

## Abbreviations

NAFLD: Non-alcoholic fatty liver disease
CVD: Cardiovascular disease
BPA: Bisphenol A
BPS: Bisphenol S
BPF: Bisphenol F
NHANES: National Health and Nutrition Examination Survey
HSI: Hepatic steatosis index
TC: Total cholesterol
TG: Triglycerides
LDL-C: Low-density lipoprotein cholesterol
HbA1c: Glycosylated hemoglobin A1c
LLOD: Lower limits of detection
HDL-C: High-density lipoprotein cholesterol
ALT: Alanine aminotransferase
AST: Aspartate aminotransferase
BMI: Body mass index
OR: Odds ratios
CI: Confidence intervals
DE: Direct effect
IE: Indirect effect
