# Supplementary material for: Serum glycolipids mediate the relationship of urinary bisphenols with NAFLD: analysis of a population-based, cross-sectional study
Source: Environ Health. 2023 Jan 2;21:124. doi: 10.1186/s12940-022-00945-w (PMC9806917; doi:10.1186/s12940-022-00945-w)
Supplement: Supplementary file 1 — Additional file 1: Table S1. Baseline characteristicsof the study population according to NAFLD and non-NAFLD stratified by sex, NHANES2013–16. Table S2. Urinary concentrations of BPA, BPF and BPS according to sex. Table S3. Associations of urinary BPA, BPS, and BPF levels with NAFLD stratified by sex. Table S4. Associations of urinary BPA, BPS, and BPF levels with NAFLD in participants with urinary BPF concentrations above the LLOD (N=525). [file 12940_2022_945_MOESM1_ESM.docx]

**Table S1. Baseline characteristics of the study population according to NAFLD and non-NAFLD stratiﬁed by sex, NHANES 2013–16.**

|  | **Female** |  |  | **Male** |  |  |
| --- | --- | --- | --- | --- | --- | --- |
|  | **Non-NAFLD** | **NAFLD** |  | **Non-NAFLD** | **NAFLD** |  |
| **Variables** | ***N=211*** | ***N=305*** | **p value** | ***N=209*** | ***N=235*** | **p value** |
| Age ^a^ | 52.8 (17.9) | 53.3 (16.2) | 0.773 | 53.2 (19.0) | 52.2 (16.4) | 0.528 |
| Sex, n(%) |  |  | - |  |  | - |
| Female | 211 (100%) | 305 (100%) |  | - | - |  |
| Male | - | - |  | 209 (100%) | 235 (100%) |  |
| Ethnicity, n(%) |  |  | <0.001 |  |  | <0.001 |
| Non-Hispanic White | 81 (38.4%) | 107 (35.1%) |  | 88 (42.1%) | 91 (38.7%) |  |
| Non-Hispanic Black | 34 (16.1%) | 80 (26.2%) |  | 39 (18.7%) | 59 (25.1%) |  |
| Non-Hispanic Asian | 52 (24.6%) | 17 (5.57%) |  | 44 (21.1%) | 16 (6.81%) |  |
| Mexican American | 17 (8.06%) | 60 (19.7%) |  | 19 (9.09%) | 34 (14.5%) |  |
| Other Hispanic | 27 (12.8%) | 41 (13.4%) |  | 19 (9.09%) | 35 (14.9%) |  |
| Education, n(%) |  |  | 0.011 |  |  | 0.065 |
| Less than high school | 38 (18.0%) | 82 (26.9%) |  | 37 (17.7%) | 53 (22.6%) |  |
| High school or equivalent | 42 (19.9%) | 73 (23.9%) |  | 38 (18.2%) | 57 (24.3%) |  |
| College or above | 131 (62.1%) | 150 (49.2%) |  | 134 (64.1%) | 125 (53.2%) |  |
| Poverty income ratio |  |  | 0.186 |  |  | 0.720 |
| <1.0 | 49 (23.2%) | 88 (28.9%) |  | 32 (15.3%) | 40 (17.0%) |  |
| ≥1.0 | 162 (76.8%) | 217 (71.1%) |  | 177 (84.7%) | 195 (83.0%) |  |
| Smoking, n(%) |  |  | 0.671 |  |  | 0.282 |
| never | 152 (72.0%) | 212 (69.5%) |  | 109 (52.2%) | 123 (52.3%) |  |
| former | 33 (15.6%) | 47 (15.4%) |  | 65 (31.1%) | 84 (35.7%) |  |
| current | 26 (12.3%) | 46 (15.1%) |  | 35 (16.7%) | 28 (11.9%) |  |
| Drinking, n(%) |  |  | 0.368 |  |  | 0.886 |
| No or unknown | 78 (37.0%) | 126 (41.3%) |  | 43 (20.6%) | 46 (19.6%) |  |
| Yes | 133 (63.0%) | 179 (58.7%) |  | 166 (79.4%) | 189 (80.4%) |  |
| Hypertension, n(%) |  |  | <0.001 |  |  | 0.006 |
| No | 133 (63.0%) | 133 (43.6%) |  | 127 (60.8%) | 111 (47.2%) |  |
| Yes | 78 (37.0%) | 172 (56.4%) |  | 82 (39.2%) | 124 (52.8%) |  |
| Diabetes, n(%) |  |  | <0.001 |  |  | <0.001 |
| No | 190 (90.0%) | 204 (66.9%) |  | 176 (84.2%) | 152 (64.7%) |  |
| Yes | 21 (9.95%) | 101 (33.1%) |  | 33 (15.8%) | 83 (35.3%) |  |
| BMI, (kg/m^2^) ^a^ | 22.9 (2.78) | 34.1 (6.71) | <0.001 | 24.6 (2.82) | 33.0 (5.80) | <0.001 |
| TC, (mg/dL) ^a^ | 194 (40.2) | 191 (38.0) | 0.390 | 181 (37.7) | 184 (41.9) | 0.456 |
| TG, (mg/dL) ^b^ | 73.0 [54.0;104] | 99.0 [67.0;148] | <0.001 | 85.0 [61.0;117] | 118 [84.5;160] | <0.001 |
| LDL-C, (mg/dL) ^a^ | 111 (34.9) | 113 (34.6) | 0.374 | 108 (32.5) | 113 (37.0) | 0.128 |
| HDL-C, (mg/dL) ^a^ | 66.6 (15.6) | 54.9 (14.0) | <0.001 | 53.5 (16.8) | 44.5 (10.0) | <0.001 |
| Glucose, (mg/dL) ^a^ | 99.7 (29.6) | 115 (42.7) | <0.001 | 105 (20.6) | 123 (43.4) | <0.001 |
| HbA1c (%) ^a^ | 5.58 (0.93) | 6.12 (1.43) | <0.001 | 5.62 (0.81) | 6.22 (1.47) | <0.001 |
| Urinary BPA, (ng/mL) ^b^ | 0.90 [0.45;1.65] | 1.20 [0.60;2.30] | 0.009 | 1.00 [0.50;2.20] | 1.40 [0.70;2.65] | 0.027 |
| Urinary BPS, (ng/mL) ^b^ | 0.40 [0.20;0.80] | 0.60 [0.30;1.40] | <0.001 | 0.50 [0.20;1.10] | 0.60 [0.20;1.20] | 0.040 |
| Urinary BPF, (ng/mL) ^b^ | 0.20 [0.14;0.60] | 0.30 [0.14;0.80] | 0.092 | 0.30 [0.14;0.80] | 0.30 [0.14;0.70] | 0.976 |
| HSI ^a^ | 31.2 (3.02) | 44.4 (7.00) | 0.001 | 31.8 (3.11) | 43.3 (6.11) | <0.001 |

^a^ data is expressed with mean (SD); ^b^ data is expressed with median [IQR]. NHANES: National Health and Nutrition Examination Survey; NAFLD: non-alcoholic fatty liver disease; SD: standard deviation; IQR: interquartile range; BMI: body mass index; TC: total cholesterol; TG: triglycerides; LDL-C: low-density lipoprotein cholesterol; HDL-C: high-density lipoprotein cholesterol; HbA1c: glycosylated hemoglobin A1c; BPA: bisphenol A; BPS: bisphenol S; BPF: bisphenol F; HSI: hepatic steatosis index, p<0.05 suggests significant differences.

**Table S2. Urinary concentrations of BPA, BPF and BPS according to sex.**

|  | **Female** | **Male** | **p value** |
| --- | --- | --- | --- |
| Urinary BPA, (ng/mL) | 1.00[0.50,2.00] | 1.15[0.50,2.60] | 0.025 |
| Urinary BPS, (ng/mL) | 0.20[0.14,0.80] | 0.30[0.14,0.78] | 0.860 |
| Urinary BPF, (ng/mL) | 0.50[0.20,1.20] | 0.50[0.20,1.20] | 0.511 |

The number of participants was 516 for female and 444 for male. BPA: bisphenol A; BPS: bisphenol S; BPF: bisphenol F, p<0.05 suggests significant differences.

**Table S3. Associations of urinary BPA, BPS, and BPF levels with NAFLD stratiﬁed by sex.**

| **Variables** | **sex** | **Tertile 1** | **Tertile 2** | **Tertile 3** | **p for**  **trend** | **p for**  **interaction** |
| --- | --- | --- | --- | --- | --- | --- |
| **BPA** | Female | 1 (ref) | 1.31(0.798,2.15) | 1.735(1.055,2.855) | 0.03 | 0.941 |
|  | Male | 1 (ref) | 1.658(0.997,2.755) | 1.769(1.047,2.988) | 0.03 |  |
| **BPS** | Female | 1 (ref) | 1.044(0.639,1.706) | 2.304(1.394,3.807) | 0.002 | 0.152 |
|  | Male | 1 (ref) | 1.566(0.937,2.616) | 1.623(0.979,2.691) | 0.054 |  |
| **BPF** | Female | 1 (ref) | 0.961(0.579,1.593) | 1.482(0.907,2.422) | 0.142 | 0.068 |
|  | Male | 1 (ref) | 1.134(0.666,1.931) | 0.932(0.563,1.544) | 0.827 |  |

Adjusted for ethnicity (non-Hispanic white, non-Hispanic black, non-Hispanic Asian, Mexican American, Other Hispanic), education (less than high school, high school or equivalent, college or above), hypertension (no, yes), diabetes (no, yes), logistic transformed levels of triglyceride, high-density lipoprotein cholesterol, glucose, glycosylated hemoglobin A1c and urine creatinine (tertiles) , p<0.05 suggests significant differences.

**Table S4. Associations of urinary BPA, BPS, and BPF levels with NAFLD in participants with urinary BPF concentrations above the LLOD (N=525).**

|  | **Tertile 1** |  | **Tertile 2** | |  | **Tertile 3** | |  | **Per Log unit increase** | |
| --- | --- | --- | --- | --- | --- | --- | --- | --- | --- | --- |
|  |  |  | **OR(95%CI)** | **p** |  | **OR(95%CI)** | **p** |  | **OR(95%CI)** | **p** |
| **BPA** |  |  |  |  |  |  |  |  |  |  |
| Model 1 | 1 (ref) |  | 1.192(0.782,1.818) | 0.414 |  | 1.192(0.782,1.818) | 0.191 |  | 1.149(0.795,1.661) | 0.460 |
| Model 2 | 1 (ref) |  | 1.211(0.758,1.934) | 0.423 |  | 1.372(0.860,2.190) | 0.184 |  | 1.130(0.752,1.698) | 0.557 |
| Model 3 | 1 (ref) |  | 1.114(0.692,1.793) | 0.657 |  | 1.135(0.694,1.857) | 0.613 |  | 0.948(0.619,1.453) | 0.807 |
| **BPS** |  |  |  |  |  |  |  |  |  |  |
| Model 1 | 1 (ref) |  | 1.233(0.809,1.879) | 0.331 |  | 1.949(1.276,2.976) | 0.002 |  | 1.546(1.137,2.101) | 0.005 |
| Model 2 | 1 (ref) |  | 1.274(0.798,2.033) | 0.310 |  | 1.961(1.228,3.133) | 0.005 |  | 1.612(1.148,2.263) | 0.006 |
| Model 3 | 1 (ref) |  | 1.287(0.800,2.070) | 0.298 |  | 1.996(1.223,3.256) | 0.006 |  | 1.633(1.145,2.328) | 0.007 |
| **BPF** |  |  |  |  |  |  |  |  |  |  |
| Model 1 | 1 (ref) |  | 0.866(0.570,1.317) | 0.502 |  | 1.126(0.733,1.730) | 0.587 |  | 1.317(0.948,1.830) | 0.101 |
| Model 2 | 1 (ref) |  | 0.788(0.493,1.257) | 0.317 |  | 1.138(0.704,1.840) | 0.597 |  | 1.281(0.887,1.850) | 0.186 |
| Model 3 | 1 (ref) |  | 0.747(0.465,1.200) | 0.228 |  | 1.035(0.635,1.688) | 0.891 |  | 1.210(0.833,1.755) | 0.317 |

Model 1 is adjusted urine creatinine (tertiles); Model 2 is adjusted for ethnicity (non-Hispanic white, non-Hispanic black, non-Hispanic Asian, Mexican American, Other Hispanic), education (less than high school, high school or equivalent, college or above), drinking (no or unknown, yes), hypertension (no, yes), diabetes (no, yes), logistic transformed levels of triglyceride, high-density lipoprotein cholesterol, glucose, glycosylated hemoglobin A1c and urine creatinine (tertiles), Model 3 is adjusted variables in Model 2 plus logistic transformed concentration of BPA and/or BPS. LLOD: lower limits of detection; BPA: bisphenol A; BPS: bisphenol S; BPF: bisphenol F; OR: odds ratio; CI: confidence intervals, p<0.05 suggests significant differences.
